# An Inquiry into the Logical Basis of the Germ Theory

**Published:** 1898-06

**Authors:** M. R. Leverson

**Affiliations:** Fort Hamilton, L. I., N. Y.


### AN INQUIRY INTO THE LOGICAL BASIS OF THE GERM THEORY.

M. R. Leverson, M. D., Fort Hamilton, L. I., N. Y.

(Paper read before the Homœopathic Union Brooklyn, April, 1898.)

For the purpose of investigating the logical basis of any theory, it is essential that that theory should be considered in the very words of its author, wherever that is possible. I therefore propose to consider the germ theory in the words of Dr. Fraenkel according to the "fixed and definite rules laid down with exactness and precision by Koch." (See *Text Book of Bacteriology*, by Fraenkel, translated by Dr. J. H. Linsley, edition of 1891, pp. 150-1.)

These rules, as stated by Dr. Fraenkel, are; that for a micro-organism to be recognized as a specific agent in the production of pathological alterations it should fulfill three conditions, as follows:

"First. It must be proved to be present in all cases of the disease in question.

"Second. It must further be present in this disease and in no other; since otherwise it could not produce a specific definite action.

"Third. A specific micro-organism must occur in such quantities and so distributed within the tissues that all the symptoms of the disease may be clearly attributed to it."

The first of these conditions constantly assumed present by the bacteriologists is true only by giving a name to a condition presenting certain symptoms, and excluding conditions which, though essentially alike, may present different symptoms; and even then the presence of such condition cannot be asserted in every instance of the finding of such micro-organism.

The error in this case arises chiefly through the use of a nosology by the old school which has little reference to the real morbid condition. The true homœopath will readily appreciate this objection, but it will have little weight with persons habituated to the prevalent system of nosology, nor is it necessary for my present purpose to insist upon it.

But the second of the above "conditions" has not been secured with regard to any of the so-called germs.

The same objection applies to the third condition, with the additional objection that it begs the whole question by assuming that symptoms may be "clearly attributed" to a specific micro-organism as a cause.

Neither the second nor third conditions has ever been attained, and from the nature of the case it seems impossible that either of them ever should be; nevertheless the bacteriologists, from Koch downwards, continually argue by assuming their existence in every case in which they have desired to set up bacterial peccancy; and yet Professor Crocq assures us that the supposed cholera bacillus is often found accompanying typhoid fever, a simple stomach ache, and even perfect health. (See his address before the Belgian Academy of Medicine, March 30th, 1895.)

Mr. Bouchadat, an advocate of vaccination, stated before the French Academy: "The system of Pasteur, that every contagious and virulent disease has for its cause a microbe, a pre-existent specific germ floating in the atmosphere or deposited on the earth, apparently true in some, fails in many well defined cases." He then specified hydrophobia, syphilis, typhus, cholera, small-pox, typhoid, yellow fever, and the plague, of which the supposed microbe has been disproved!

But even if all the conditions prescribed by Koch's three rules were complied with, they would be insufficient, logically, to establish the conclusion drawn from their supposed presence. It must be remembered that the inoculation of so-called germs into a human or other living body is an entirely different process from that which occurred when they have been found in a condition of disease. The conditions are so entirely different that one is at a loss to account for their confusion. But, further, even the inoculation is defective. These supposed germs being cultivated in certain media, are so inseparably intermixed with their respective cultures and the poisons produced therein, that it is impossible to determine whether the so-called germs or their accompaniments produce the diseased conditions that follow the inoculation. Koch, Pasteur, Fraenkel, DeBary, Pruden, and their numerous disciples must add another rule to the "three fixed and definite rules laid down with exactness and precision by Koch" before any, even a mere tyro, in logical reasoning can accept their conclusions. Not only must "the germ in question be proved present in all cases of the disease in question," according to the second of the Kochean rules, but all other sufficient causative matters and conditions must be absent.

This no manipulation has been able to effect, and all that bacteriology has really shown is that certain so-called germs do often accompany certain diseases, but whether as cause or effect remains at present unknown, with a great preponderant probability in favor of the latter, while as Professor Crocq has shown they are often present in the organism without any disease.*

An example almost daily presented to the practicing

---

*In *Origines Épidémiques*, a work I have only just come across, (Nancy A. Nicolle, publisher, 1896,) Dr. H. Boucher, of Saint Servain, France, completely overthrows the "germ theory." He shows that the so called germs are almost always, when present, the *products*, not the causes of disease, and that organic fermentation together with climatic conditions, acting on susceptible cases are the true causes of disease.—M. R. L.

physician ought to guard him against coming to any conclusion in favor of the bacterial origin of disease.

One or other form of the staphylococcus or streptococcus has always been found in pus; so much so, that Fraenkel has been led to assert, "The fact that the staphylococcus is not a regular and harmless concomitant of purulent inflammatory processes, but their cause, has been demonstrated (?) by successful transmissions." (Fraenkel's *Bacteriology*, by Lindsley, p. 323.) Yet two pages earlier (p. 321) he stated: "The investigations of Scheurlen, Steinhaus, Kaufman, and especially Gravity and De Bary, leave us no longer in doubt as to the fact that many germ-free chemical substances (such as Nitrate of Silver, Oil of Turpentine, Liquor Ammonia, Caustici, Digitaline, Cadaverine, etc.) can produce an acute suppuration in the subcutaneous tissue."

Now in the very common occurrence of sporadic cases of boils and carbuncles, without any other apparent diseased condition, if these germs are the cause thereof, how did they get into the body to produce suppuration? If, as alleged by some, they are always present in the air, what determines them to cause suppuration once in a while, in this or that individual, while at other times he feels no ill effects? This reasoning is specially forcible with regard to the deeply seated carbuncle; and why the staphylococcus should cause a boil to-day, or in one person, and a carbuncle at another time or in different persons, or why sometimes anthrax should be present with carbuncle and only the staphylococcus or streptococcus at other times, are questions which no bacteriologist has attempted to answer; and until full answers are given to these questions, the theories of Koch, Pasteur, and their followers remain the barest assumptions.

Nor should it be forgotten that with regard to many of the so-called germs, evidence is lacking to prove that they possess life or are capable of reproduction, either by sporulation or segmentation.

M. Chavée Leroy, of Claremont (France), and Dr. Ed. Fournié, the learned editor of the *Revue Medicale* of Paris, have raised grave doubts upon these questions.

As occurring in fact then in inoculations the so-called germs are intermixed inseparably with other things unknown both as to quality and quantity and, so intermixed, are injected by, or inoculated into living beings.

Let us now reduce the theory of the bacteriologists, as by them applied in practice to the form of a syllogism, and by so doing the real logical basis of that theory will be more readily perceived.

Certain germs under certain conditions (*i. e.*, such as just mentioned) produce certain diseases;

Or, some A's are B's.

Such diseases occur under these conditions, and under other (unknown) conditions;

Or, some B's are C's.

Therefore, all such diseases are caused by the germs, or all C's are A's!

In view of such logic would it not be in order to require candidates for the degree of doctor of medicine to pass a satisfactory examination in the art of reasoning as a condition of graduation, if not even of entering a medical college? At least,—that is—if any examination is to be insisted on as preliminary to the practice of the art of healing.

A story goes, that an old Irish doctor who had diligently studied all the chemisal, physiological, and biological medical theories of his day, accepting with ardor every new theory as it arose, had all his convictions disturbed by the rise of the germ theory, which he was unable quite to accept. Walking one day on the road to Limerick, his Olfactory organs were assailed by the odoriferous emanations which, with a revival of his youthful love of investigation, he soon traced to the decomposing carcass of an ass. Following up the bent given to his mind in youth, he at once resolved on pursuing a closer investigation, and he soon discovered that the cadaver teemed with *maggots.* At once his mind was illuminated, his past doubts were swept away, the germ theory in all its beauty became clear to him as a revelation! "Poor baste," said he, compassionately, "sure 'tis aisy to see what ailed him; 'tis the *maggots* kilt him sure!"

History is silent as to the Medical College from which our hero graduated!

The animal man, the physical body, needs to be supplied with air, water, food, exercise and sleep; without a supply of each of these life can be sustained only for a short period. These may all be supplied, but unsuitable in quantity or quality. The food may be bad food, or deficient or excessive in quantity; the water may be unwholesome; the air may be impure, the exercise may be insufficient or excessive; rest and sleep may likewise be so. The human being subjected to any of these defective conditions cannot be healthy. He may grow, he may become adult, but his growth is unhealthy, and he develops a weakly constitution. He is in a diseased condition, ready to fall a prey to any noxious influence which may present itself. It is probable that then may be produced or developed in him the bodies which the skill of the microscopist has detected, and to which with singular lack of logic he has ascribed miraculous causative powers; being in part seduced into this bad logic by the practice of giving names to diseases as though each was a specific entity, the folly of which was, I believe, first laid bare by the illustrious Hahnemann. The deficient, excessive, or ill-adapted food, water, air, exercise, or rest, impoverishes the blood, which thus fails to nourish the organs of the body, whereby these are rendered incompetent to perform their allotted functions. The organs of secretion and of excretion act imperfectly, and the blood becomes loaded with poisons which the proper organs had failed to eliminate. Now nature makes a supreme effort and seeks to get rid of the poison through the skin by means of one of the exanthems. One of these, and generally, as was truly said by Sydenham the mildest and safest, is small-pox, which acts as a safety-valve; as evidenced by the fact that years of greatest small-pox are usually years of least mortality. If, then, these conditions be got rid of, which tend to impair the action of the various organs of the body, the real cause of disease is removed. Thus science, after divarications innumerable by her pseudo followers, brings us back to the plain teachings of common sense, viz., that the only true prophylactic against any and all disease is health, and the removal of the conditions which weaken vitality is the only mode by which health can be preserved.

Investigation into the logical basis of the germ theory is thus seen to show THAT THERE IS NONE!

But let me here guard against an erroneous conclusion which some might draw from the foregoing argument.

While there is absolutely no demonstration of the causative function of germs, nor even of probability in the germ theory, neither is the contrary proved or asserted.

In the absence of knowledge upon the subject possessed, not merely by the ordinary physician, but by the highest real or supposed authorities, the only course open to the true man of science is "to hold his judgment in suspense." The building up of medical practice on the assumption of such theory is unwarranted—its dogmatic enforcement is a crime.

